# Therapeutic Notes

**Published:** 1899-02

**Authors:** W. J. Martin

**Affiliations:** Kankakee, Ill.


### THERAPEUTIC NOTES.

UNDER THE DIRECTION OF W. J. MARTIN, M.D.C.,
KANKAKEE, ILL.

IODINE IN PURPURA HEMORRHAGICA.—The treatment consists of intratracheal injections of iodine crystals and iodide of potassium and one-half ounce of distilled water, sufficient to dissolve the chemical compounds. The proportions are: ℞.—Iodine cryst., gr. x; kalii iodi., ℈ij; aqu. dist., ℥iv. M.—et. fiat. injectis. Sig.—One dose for intratracheal use. I inject this quantity morning and night for three days; then once a day, gradually decreasing amount of dose till œdema subsides, and the petechial spots in the nostrils disappear, the continued use depending upon the severity of the attack and the condition the individual presents to the veterinarian. I also employ strychnine, to be given by the mouth, in the form of a solution, such as ℞.—Strychnine, grs. ij; aq. dist., ℥ij.—M. Sig. ℥ij on tongue every three hours. The frequency of the dose depends upon the condition of the animal, such as debility, exhaustion, prostration, anorexia, etc. If the strychnine be boiled in water, after which a small amount of alcohol be added, there need be no fear of a precipitation forming or a deterioration in strength. —*G. J. G., in American Veterinary Review.*

THE administration of iodide of potassium in drachm doses morning and evening in the drinking water, is of decided benefit to assist the healing process of poll-evil and fistulous withers after operation.

ARECOLINE IN LAMINITIS.—The hypodermatic use of arecoline is at the present time having an extensive use among the veterinarians of Continental Europe in the treatment of acute laminitis, and, according to the latest authentic clinical reports, this drug is giving very satisfactory results in relieving and curing this always painful and often dangerous affection of horses. Recoveries are reported as having occurred after the use of this drug in from three to ten days. The form of the drug employed was the bromohydrate, and it was given once a day in doses ranging from 5 to 10 centigrammes dissolved in 5 grains of distilled water.

Treatment of Cancer.—The treatment of cancer and cancerous neoplasms by the hypodermatic injection of alcohol has been again revived in human practice after a lapse of nearly twenty years. The experimenters were Hasse and Kuh. The former claimed to have cured fifteen out of eighteen cases of mammary cancer ; the latter claiming to have cured a case of primary cancer of the nasopharyngeal region by the same method. About one-third of absolute alcohol to two-thirds of distilled or boiled water is the proper method of preparing this injection. This is injected twice a week into the growth. The amount of fluid injected varies according to the size of the neoplasms, from ten to twenty Pravaz syringefuls being the dose for an adult person.. It is claimed by the experimenters that the manner in which the treatment cures cancer is by forming a connective-tissue capsule around each growth, causing obliteration of the bloodvessels and contraction of the neoplastic tissues. This method of attacking malignant neoplasms might be successfully employed in veterinary practice when their removal by surgical procedure was contraindicated.

E.C. P. Anæsthetic Mixture.—Schleich (*Report de Pharm.*), as the result of extended experiments, suggests a combination of ether with chloroform and petroleum ether as a safer anæsthetic. He gives the varying proportions as follows : No. 1. Ether, 180 parts ; chloroform, 15 parts ; petroleum ether, 15 parts. No. 2. Ether, 60 parts ; chloroform, 30 parts ; petroleum ether, 15 parts. No. 3. Ether, 15 parts ; chloroform, 15 parts ; petroleum ether, 15 parts.

Anodyne Liniment.—℞.—Lini. sapo. co., ℥vj. ; lini. camphoræ co., ℥vj. ; tr. opii, ℥vj. ; lini. belladonnæ, ℥j; sol. ammonia fort., ℥j.—M. A useful embrocation for bruises, rheumatoid arthritis, etc.

Vehicle for Iodine.—When it is desired to administer iodine in solution by the mouth, a convenient vehicle will be found in cow's milk.

FOR MASTITIS.—As an adjuvant to other proper treatment, the following is a most serviceable application to the udder of mares and cows suffering from this most painful disease: ℞.—Tr. belladonna folia, sp. camphoræ, ol. olivæ, āā equal parts. The mixture may be applied several times daily.

COMPOUND ALUM POWDER (SQUIBB'S).—℞.—Gum camphor, 2 parts; carbolic acid, 4 parts; exsiccated alum, finely pulverized, 94 parts. Dissolve the camphor in the liquefied carbolic acid, then incorporate with the alum. A useful dusting powder in skin abrasions.

TO DISGUISE ODOR OF FORMALDEHYDE.—A writer in the *Phar. Zeitung* says that the odor of formaldehyde may be successfully disguised by preparing the mixture as follows: 25 parts of formalin, 5 parts of tincture of eucalyptus, alcohol enough to make 200 parts. A teaspoonful represents 0.025 grammes of pure formalin.

CREOSOTE PILLS.—Delhaaxhe's formula is as follows: ℞.—Creosote, 10 parts; water, 5 parts; licorice root, 20 parts. M.—*Western Druggist.*

SOLUTION FOR CONJUNCTIVITIS.—In that form of conjunctivitis so commonly seen in horses in which the lachrymal discharge is profuse and the pain great, the following lotion, applied after first fomenting the eyes with warm water, will be found of benefit: ℞.—Plumbic acetat, grs. viij; zinci. sulph., grs., viij; morphia sulph., grs. iv; aq. dist., ℥iv.—M. Sig.—Five or six minims may be dropped into the eye several times a day, or it may be applied with a feather.

PURULENT CONJUNCTIVITIS.—Iodoform is also useful in treating this disease. The drug is best used in the form of an unguent, thus: ℞.—Iodoform, ʒj; lanolin, ʒiv.—M. This may be smeared around the inner surface of the lids by means of a camel's-hair brush or a feather.

ONE part of aloin is equivalent to four parts of Socotrine aloes.

ANTISEPTIC SOLUTION.—℞.—Hydrarg. biniod. rubri, potassi iodid, āā grs. xv; aq. dist., Oij.—M. 1 to 1000. Solution, a powerful antiseptic and germicide.

